# Employing DNA binding dye to improve detection of *Enterocytozoon hepatopenaei* in real-time LAMP

**DOI:** 10.1038/s41598-019-52459-0

**Published:** 2019-11-01

**Authors:** Biao Ma, Huanteng Yu, Jiehong Fang, Chuanxin Sun, Mingzhou Zhang

**Affiliations:** 1 Zhejiang Provincial Key Laboratory of Biometrology and Inspection & Quarantine, China Jiliang University, Hangzhou, 310018 China; 2 Department of Plant Biology, Uppsala BioCenter, Linnean Centre for Plant Biology, Swedish University of Agricultural Science (SLU), P.O. Box 7080, SE-75007 Uppsala, Sweden

**Keywords:** Parasitology, Assay systems

## Abstract

*Enterocytozoon hepatopenaei* (EHP) is a pathogen in the pancreatic tissue of prawn, as reported in recent years. Enterosporidiosis caused by EHP in Penaeus genus is spreading widely, which seriously threatens the sustainable development of shrimp aquaculture in the world. Empolying the DNA binding dye of SYTO-16, a real-time quantitative loop-mediated isothermal amplification (LAMP) method has been established herein to detect EHP. The primer sequences used in the LAMP reaction were according to the SSU rRNA gene. The LAMP assay has reached a sensitivity of 10^1^ copies/µL and no cross-reaction with other aquatic pathogens. Compared with normal PCR, the efficiency of the LAMP technique is more sensitive, which has a shorter detection time. The use of fluorescent nucleic acid dye (SYTO-16) reveals a more satisfactory performance relative to calcein. The quantitative LAMP assay can be considered as a valid tool for rapid detection of microsporidian in prawns. Our study provides a scientific basis to follow the effect of the pathogen infection on growth of cultured penaeid shrimp.

## Introduction

In recent years, infection of *Enterocytozoon hepatopenaei* (EHP) in *Penaeus vannamei* had been widely spread in the Asia-Pacific region^1^. The pathogen was first discovered and isolated in Thailand in 2009 in the cytoplasm of the host hepatopancreas tubule epithelial cells^2^. In China, the infection of EHP was detected in cultured prawns as early as 2013. Although the pathogen did not lead to the death of prawn, it slowed down dramatically the growth of penaeid shrimp. An infected shrimp could continue to survive and feed, but the growth of the shrimp was slow or even stagnant. In 2015, the infection rate in examined samples of cultured shrimp was about 25% in Jiangsu province, resulting in a 15–20% reduction in breeding output. More than half of the farmers suffered losses, resulting in a loss of 300 million^3^. To mitigate the impact of disease outbreak and benefit the aquaculture industry, it should be of high importance to develop a highly effective method for early detection of EHP in shrimp.

The size of the microsporidian was too small to detect by a conventional optical microscopic examination. Over the past several decades, several reliable and powerful molecular diagnostic techniques such as polymerase chain reaction (PCR)^2^, nested PCR^4^ and quantitative PCR (qPCR)^5^ have developed to trace the pathogen. However, these methods require fully equipped laboratories with good infrastructure, reliable electrical supply, and highly trained staffs. Due to those reasons, various methods of the isothermal amplification of nucleic acids have been developed^6^. The loop-mediated isothermal amplification (LAMP) assay was an excellent diagnostic tool because of its simplicity, cost-effectiveness, high efficiency, and specificity^7^. This method needed a four-primer set, designed to recognize six distinct regions on the target gene, and required the enzyme *Bst* polymerase which had strand displacement activity. In addition, loop primers could be added to the assay reaction which was designed according to the four primers set to enhance efficiency and increase specificity of the assay^8^.

The major benefit of LAMP was to amplify nucleic acids without a need of expensive laboratory equipment. In a laboratory, an expensive temperature cycling machine and a real-time measurement of product application are usually required. In the case of LAMP, the cycling machine is no longer required and measurements can be of turbidity, fluorescence, ion concentrations, and color for visualization of product amplification^9^. For the fluorescent measurement, several types of specific probes, intercalating dyes, and calcein were tested for detection. Suebsing *et al*. established a LAMP assay by using colorimetric nanogold for detection of microsporidian at 65 °C^10^. The assay needed approximately 50 minutes^10^. Other approaches of amplification such as real-time LAMP technology were also performed to optimize amplification and lower detection limitation of EHP in shrimp^11^. For example, ten copies were the detection limit for a LAMP protocol while using a closed tube system and SYBR™ green I dye^12^. Different fluorescent dyes have been compared for a laboratory qPCR^13–16^. Only few studies, however, have been done to compare different intercalating dyes in quantitative LAMP^9,17^.

We aim at finding a suitable fluorescent dye for use in quantitative LAMP, which could not only provide better efficiency and sensitivity, but also break through the bottleneck of non-quantitative LAMP. Furthermore, the best combination of time-to-threshold and signal-to-noise ratio (SNR) were taken into account as one of the key parameters. Considering the environment of on-site testing of actual samples, the method might be used on cheaper, mobile and easier deployable equipment. The established quantitative LAMP assay by using SYTO-16 appears to be an accurate and sensitive method for rapid detection of the pathogen in shrimp and for early diagnosis and intervention in shrimp aquaculture.

## Results

### Optimization and reaction

**Figure 1:**
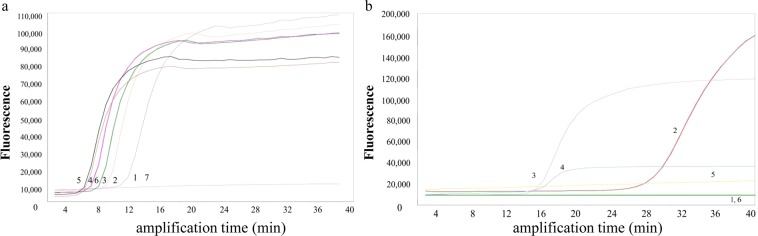
Optimization of reaction temperature and dye concentration for fluorescence quantitative LAMP assay. (**a**) 1–6: 60 °C, 61 °C, 62 °C, 63 °C, 64 °C and 65 °C, 7: No template control. (**b**) 1–5: The final concentration of SYTO-16 with 1 mM, 0.1 mM, 0.01 mM, 0.001 mM and 0.0001 mM, 6: No template control.

In order to determine the optimal conditions of fluorescence-quantitative LAMP, the positive standard plasmids were used as the target template. The fluorescence-quantitative LAMP assay was conducted under isothermal conditions between 60 °C and 65 °C. No significant difference was observed when the temperature was higher than 65 °C (Fig. 1a). For fluorescence-quantitative LAMP reactions performed at 65 °C for 5–60 minutes, the amplification products could be observed first at 8 minutes and then more clearly at 20 minutes. Subsequently, there was no change in fluorescent value when the time was more than 40 minutes (data not shown). To improve time-to-threshold and SNR, we optimized the concentration of reagents and parameters of reaction. The reaction was conducted with different concentrations of fluorescent dye, ranging from 0.0001 to 1 mM. According to the amplification time, the final concentration of SYTO-16 was determined as 0.01 mM (Fig. 1b).

### Analytical sensitivity and specificity of fluorescence-quantitative LAMP

**Figure 2:**
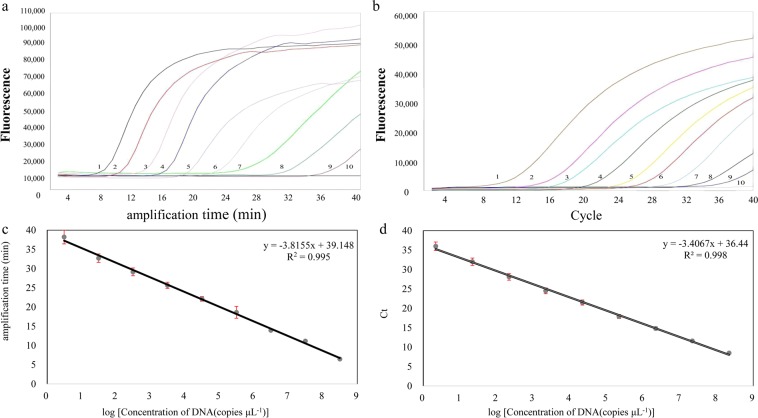
Sensitivity of quantitative LAMP assay and qPCR assay for *Enterocytozoon hepatopenaei*. 1–9: 2.238 × 10^8^, 2.238 × 10^7^, 2.238 × 10^6^, 2.238 × 10^5^, 2.238 × 10^4^, 2.238 × 10^3^, 2.238 × 10^2^, 2.238 × 10^1^, 2.238 × 10^0^ copies/µL, 10: No template control.

To evaluate the sensitivity of detection, the standard plasmid was serial diluted with TE buffer by 10 times. The range of 2.238 × 10^8^ to 2.238 copies/µL were added to the corresponding tube as templates to determine the detection limits. Every test was repeated three times. To ensure the reliability of these experimental data, the qPCR assay was used as a reference. The result showed that the detection sensitivity of fluorescence-quantitative LAMP method was 10^1^ copy numbers of pMD19-EHP plasmid DNA, in contrast to the same detection limit of real time quantitative PCR (Fig. 2a,b). The amount of time needed to develop a visual fluorescence value unit in these experiments was inversely proportional to the amount of template, demonstrating that the signal is quantitatively proportional to the abundance of template^18^. The results of the standard curves indicated that there were significant correlations (R^2^ = 0.995) between the amplification time and the template concentration (Fig. 2c,d).

**Figure 3:**
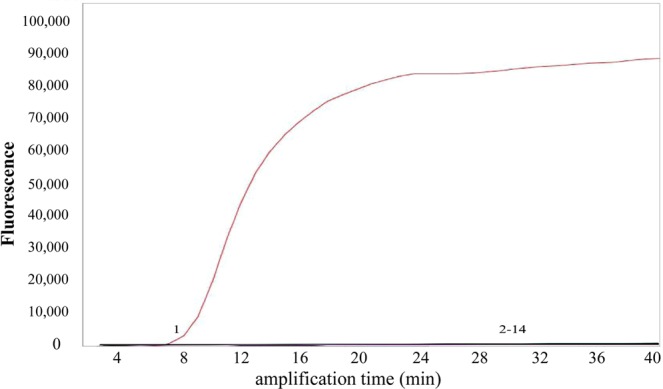
Specificity of the quantitative LAMP assay. 1–13: EHP, WSSV, IHHNV, Vibrio harveyi, Vibrio fluvialis, Vibrio cholerae, Shigella, Salmonella typhimurium, Streptococcus mutans, Bacillus subtilis, Escherichia coli, Vibrio parahemolyticus, Staphylococcus aureus, 14: No template control.

In order to verify the specificity of the primers of EHP, 12 other pathogens were carried out by fluorescence-quantitative LAMP assay. The high specificity of the fluorescence-quantitative LAMP assay was provided by the use of three primer sets (F3/B3, FIP/BIP and LF/LB), all of which were designed in the way of that only perfect matches could lead to amplifications. All samples used in specificity were tested in 3 replicates. As expected, only the samples represented to the EHP genomic DNA, had significant changes in fluorescence with real-time instrument (Fig. 3). As a comparison, the qPCR assay also showed the same results. Therefore, it confirmed that the primers were highly specific.

### Comparison of sensitivity between fluorescence-quantitative LAMP and qPCR assays to EHP detection in infected samples

**Figure 4:**
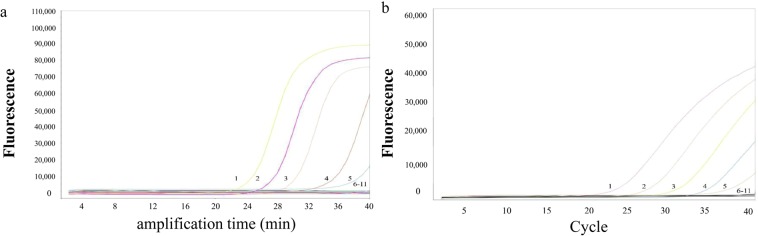
Sensitivity of quantitative LAMP and qPCR assays for EHP detection in actual samples. 1–10: 1.78 × 10^1^, 1.78 × 10^0^, 1.78 × 10^−1^, 1.78 × 10^−2^, 1.78 × 10^−3^, 1.78 × 10^−4^, 1.78 × 10^−5^, 1.78 × 10^−6^, 1.78 × 10^−7^, 1.78 × 10^−8^ ng/µL, 11: No template control.

In the practical application of detection, the most of samples contained low quantity DNA. The high sensitivity was important and necessary for rapid diagnosis in place. In the experiment, the genomic DNA gave a reasonable result in sensitivity and linearity of the fluorescence-quantitative LAMP technique with the initial concentration as 17.86 ng/µL. The ratios of 260/280 and 260/230 were 1.64 and 0.86, respectively. Serial dilutions of the infected sample, and the final concentrations were ranged from 1.78 × 10^−8^ to 1.78 × 10^1^ ng/µL. The decreasing concentration indicated that the detection limit for the infected sample by fluorescence-quantitative LAMP assay were 1.78 × 10^−2^ fg (Fig. 4a). The qPCR could also detect the same orders of magnitude (Fig. 4b). The detection limit of fluorescence-quantitative LAMP assay was on the same order of magnitude as previous studies^10^.

### Tests of practical samples

The applicability of fluorescence-quantitative LAMP assay for 116 blind samples was demonstrated. There were 36 positive samples detected and the positive rate was 31.03%. All the samples were also analyzed by qPCR assays as a standard reference. There were 35 positive samples detected and the positive rate was 30.17%. The concordance between fluorescence-quantitative LAMP assay and qPCR tests in 116 blind samples for detection of the EHP were shown (Table 1). The results of the two methods are consistent with the rate of 96.9%.

**Table 1:** Concordance between quantitative LAMP and qPCR tests in specimens for detection of EHP.

Test result	Numbers of samples (%) with qPCR		Total numbers of samples (%)	Absolute agreement (%)	Kappa value (95% CI)
	EHP(+)	EHP(−)			
**quantitative LAMP result**					
EHP(+)	34	2	36 (31.03)	96.9	0.94
EHP(−)	1	79	80 (68.97)		
Total	35 (30.17)	81 (69.83)	116		

CI, confidence interval.

EHP(+) result refers to EHP detection, EHP (−) result reflects the absence of EHP.

## Discussion

In the past decades, there were many several reliable and powerful technologies available for the detection of EHP genomic DNA. The most widely used method for detection of EHP infection was based on the molecular level, such as PCR. However, this method might not be suitable in developing countries or for field use, because of requirement of sophisticated instrumentation, complicated assay procedures, expensive reagents and well-trained staffs^19–21^. Therefore, an expanding variety of novel nucleic acid amplification technologies has been developed to meet the challenges of performing diagnostics without well-equipped facilities in low resource settings. With this focus, many nucleic acid detection methods have been developed under isothermal conditions. Some of these methods have been shown to detect a small number of copies of nucleic acids^22^. Among these isothermal methods, LAMP assay is an excellent diagnostic tool. It provides an attractive option due to its simplicity, cost-effectiveness, high efficiency, and specificity. Under isothermal conditions, the assay can be completed using a water bath or heating block, which are readily available in an electricity-accessible condition.

Despite remarkable specificity, this method has the shortcoming of post-incubation contamination once the tubes are opened. In order to reduce the risk of pollution and eliminate the need to open the tubes, the fluorescence dyes offer greater flexibility and less cost. The tube lid need not be opened to eliminate the aerosol pollution for excluding the false positive results^23^. When template sequences vary, dye-based detection helps prevent false negatives that might result from base-pair mismatches^14^. As long as optimal concentration of fluorescent dye was determined, the method gave the best results of short amplification time, high fluorescence and low inhibition. Signal-to-noise ratio (SNR) is an important character that is defined as the ratio of the signal intensity to the noise intensity. A high SNR means a high reliability of signal and a low probability of false-positive results. In previous studies, the SNR was calculated as the ratio of fluorescence in the completed reactions with DNA template compared with the no-template control^9^. According to the rule, SYTO dyes demonstrated the best SNR at appropriate concentrations tested and gave better results than other fluorescence dyes^14,23^.

In this study, the LAMP assay combined with fluorescence dye (SYTO-16) for the detection of EHP was simple and effective for specific detection of the SSU rRNA gene under optimized conditions at 65 °C for 40 minutes. The amplification time was defined as the moment of produced fluorescence. Based on this, the amplification was observed from 8 minutes, the complete reaction duration was optimized to 40 minutes. The set of loop primers, which increased the efficiency of amplification and made the detection more rapidly^23^. The concentration of fluorescence dye were optimized because these can influence the SNR as previously described. It was found that 0.01 mM brought the best combination of time-to-threshold and signal-to-noise ratio in the LAMP reaction. Compared with the traditional non-quantitative LAMP method, the established assay could break through the limitations to achieve the quantitative detection of EHP in real time. Moreover, amplification of the short target fragment (<300 bp) was not affected by betaine, which reduces nonspecific amplification^10^.

The LAMP method was able to detect EHP in as little as 1.78 × 10^−2^ fg total DNA extracted from infected shrimp. In consideration of the sensitivity test with the standard plasmids, the calculated copy number of the plasmid obtained by the formula conversion is the same as the detection limits of the infected sample concentration. The two data were in the same orders of magnitude by calculation. As for the difference of coefficient, the main reason may be due to the purity of templates. The experimental results show that the purity of nucleic acid directly affects the amplification efficiency (Table S1). Meanwhile, there is a linear correlation between the genome quantity and amplification time in the LAMP method. The amount of time needed to develop a visual fluorescence value unit in these experiments was inversely proportional to the amount of template, demonstrating that the signal is quantitatively proportional to the abundance of template^18^. The result of the standard curves indicated that there were significant correlations between the amplification time and the template concentration (R^2^ = 0.995). In addition, no cross-amplification with other pathogens was found, indicating that the assay was highly specific for the detection of EHP.

The applicability of the fluorescence-quantitative LAMP assay was assessed with field samples, and the results were compared with those obtained from the qPCR method. It showed that in 116 blind samples, there were 34 same positive samples by using the fluorescence-quantitative LAMP and qPCR, the positive detection rates were 29.31%, which were in agreement with the determination from ZheJiang Entry-exit Inspection and Quarantine Bureau. The results of the two methods are consistent with each other (a match of 100%).

Taking together, the method is time-saving with high specificity, sensitivity, accuracy and repeatability. Importantly the method does not require a laboratory facility. After experiment (Fig. S2), the quantitative LAMP assay could be applied on the portable constant temperature fluorescence detecting instrument (Gene-8C, Allsheng Instruments Co. Ltd., Hangzhou, China). When combined with the simple and fast DNA extraction method, the method would be potentially useful for EHP detection on site and also in the laboratory to save time and costs.

## Materials and Methods

### Preparation of shrimp samples

To make templates for specificity tests, the fresh cultured shrimp samples were collected from a local aquatic product market, and natural shrimp samples were obtained from local aquafarm. The infected shrimp samples used in the present study were provided by Zhejiang Mariculture Research Institute. In addition, a total of 116 blind samples were also provided by ZheJiang Entry-exit Inspection and Quarantine Bureau.

### DNA extraction and recombinant plasmid construction

Total hepatopancreatic was extracted from 50 mg of infected shrimp hepato-pancreas using a commercial kit (TIANamp Marine Animals DNA Kit, TIANGEN, China) according to the manufacturer’s protocol. Quantity and quality of the extracted DNA was determined by measuring the ratio of A260/A280 on a micro spectrophotometer (Nano100, ALL SHENG, China). Extracts were stored at −20 °C until use.

To evaluate analytical sensitivity and specificity of the EHP assay, a standard plasmid was used as positive controls. The purified DNA and primers (listed in Table 2) were used to construct the standard plasmids. Each pair of primers, designed by using Primer Premier 5.0 software (Premier Biosoft, Canada), were dissolved in ddH_2_O to a concentration of 10 mM and added to the PCR system (Promega, Madison, USA). All reaction mixtures were prepared in a final volume of 20 μL containing 5 μM of each primer, 3 μM of dNTPs, 30 μM Mg acetate, enzymes and 10 × reaction buffer, and 1 μL DNA. The thermal cycle program was: 95 °C for 2 minutes, followed by 32 cycles of 94 °C for 30 seconds, 58 °C for 30 seconds, 72 °C for 30 seconds and a final extension step of 72 °C for 5 minutes. The products were then detected by electrophoresis and purified by gel-purification using the MiniElute Gel Extraction Kit (Genebase Bioscience, GuangZhou, China). The purified target fragments were ligated into the pMD19-T simple vector (TaKaRa Biomedical Technology Co., Ltd., Beijing, China) by using DNA Ligation Kit Ver.2.1 (TaKaRa Biomedical Technology Co., Ltd., Beijing, China), and the ligated product was transformed into *Escherichia coli* DH5a using a standard procedure. Recombinant plasmids were confirmed by PCR and sequencing. Plasmids were extracted from *E. coli* DH5a and used as standard plasmids for assays. Concentration of the recombinant plasmid is converted to copy numbers based on the following equation: Number of copies = (M × 6.02 × 10^23^ × 10^−9^)/(n × 660), in which M is the amount of DNA in nanogram, n is the length of the plasmid, and the average weight of one base pair is assumed to be 660 Da.

**Table 2:** Sequences of *Enterocytozoon hepatopenaei* LAMP primers and qPCR primers/probe.

Primers	Sequence (5′-3′)	Target gene	Fragment length (bp)
PCR Primers		*ssu* rRNA	804
EHP-F	GATGCTTGGTGTGGGAGAA		
EHP-R	CCCCCCATCAATTTCCAACG		
LAMP Primers			191
EHP-F3	TTTCGGGCTCTGGGGATA		
EHP-B3	CCCCCATCAATTTCCAACGG		
EHP-FIP	AAGCAGCACAATCCACTCCTGGTTTTGCTCGCAAGGGTGAAACT		
EHP-BIP	AACGCGGGAAAACTTACCAGGGTTTTGCACCACTCTTGTCTACCTC		
EHP-LF	GTCCTTCCGTCAATTTCGCTT		
EHP-LB	TCAAGTCTATCGTAGATTGGAGACA		
qPCR Primers			160
EHP-FP	GCTGTAGTTCTAGCAGTA		
EHP-RP	GCGTTGAGTTAAATTAAGC		
EHP-Probe	CCTGGTAGTGTCCTTCCGTCAAT		

F refers to forward and R refers to reverse.

### LAMP primers and establishment of fluorescence quantitative LAMP assay

Template sequences (Fig. S1) were obtained from GenBank in NCBI database (accession numbers: SSU rRNA gene, FJ496356). After aligning by using Clustal W software, the specific regions were selected as the target fragment. Based on the detailed analysis and comparison, specific primers (Table 2) of LAMP including two loop-primers were designed with the online tool Primer Explorer V4.0 (http://primerexplorer.jp/e/), which was supplied by Eiken Chemical (Tokyo, Japan). All primers were synthesized by Invitrogen (ThermoFisher Scientific, Waltham, USA).

The DNA template of 2.5 µL was added to the amplification reagent (total volume 25 µL) containing 10 µM outer primers (F3 and B3), 80 µM inner primers (FIP and BIP), and 40 µM loop primers (LF and LB), together with 20 mM Tris-HCl (pH 8.8), 10 mM KCl, 8 mM MgSO_4_, 10 mM (NH_4_)_2_SO_4_, 10% Tween20 (Sigma-Aldrich, St. Louis, USA), 0.8 M betaine, 0.5 mM MnCl_2_, 1.4 mM dNTPs (ThermoFisher Scientific, Waltham, USA) and *Bst* 2.0 DNA polymerase (NEB, MA, USA). No-template control reactions contained distilled water instead of template DNA. The DNA binding dye of SYTO-16 was purchased from Invitrogen (ThermoFisher Scientific, Waltham, USA), and the reaction fluorescent dye concentration was optimized following the monitor by using the instrumentation ABI Step One Plus™ (ThermoFisher Scientific, Waltham, USA) for fluorescence quantitative assay. The amplification program was 65 °C for 41 minutes with fluorescence data acquisition every 1 minute.

### qPCR primers and establishment of qPCR assay

A TaqMan-based qPCR assay was also established by targeting the SSU rRNA gene. Primers and a probe sequence (Table 2) were designed by Beacon Designer 7.9 software (Premier Biosoft, Canada). These primers and probes were synthesized by Invitrogen Biotechnology Co., Ltd (ThermoFisher Scientific, Waltham, USA). The reaction mix (TaKaRa Biomedical Technology Co., Ltd., Beijing, China) contained 2.5 μL DNA template, 0.2 μL RoxII, 0.8 μM of primer sets, 0.1 μM of probe, and 10 μL 2 × Premix Ex Taq (Probe qPCR), and ddH_2_O to a final volume of 20 μL. The thermal cycle program was: 95 °C for 5 minutes, followed by 40 cycles of 95 °C for 10 seconds, and 60 °C for 30 seconds.

### Detection limit and cross-reactivity of fluorescence quantitative LAMP assay

To assess the quantitative sensitivity of fluorescent LAMP assay, 10-fold serial dilutions of plasmid standard was prepared as template. The analytical sensitivity was tested using the EHP quantitative plasmid standard in a range from 10^8^ to 10^0^ copies per reaction. The amplification performance of fluorescence-quantitative LAMP was compared with real-time PCR. The threshold time was plotted against the log values of the detected molecules to generate an amplification standard curve. All experiments in this study were carried out in 3 replicates.

The specificity of the fluorescence-quantitative LAMP assay was assessed using DNA extracted from shrimps infected with 12 other pathogens following the incubation protocol, containing *White spot syndrome virus*, *Infectious hypodermal and hematopoietic necrosis virus*, *Vibrio harveyi*, *Vibrio fluvialis*, *Vibrio cholerae*, *Shigella*, *Salmonella typhimurium*, *Streptococcus mutans*, *Bacillus subtilis*, *E. coli*, *Vibrio parahemolyticus*, *Staphylococcus aureus*. If the fluorescent signal detected was less than the negative control, cross-reactivity was defined as negative.

### Evaluation of fluorescence-quantitative LAMP assay with blind samples

The infected shrimp samples were afforded by Zhejiang Mariculture Research Institute. A total of 116 blind samples were detected by fluorescence-quantitative LAMP and qPCR assays, which were provided by ZheJiang Entry-exit Inspection and Quarantine Bureau. Data collected from the two assays including standard curves were analyzed by ABI Step One Plus™ and Microsoft Excel software (Microsoft Inc., USA). Results of qPCR were judged as positive ones when the Cq value ≤ 35.

### Ethical approval

This article does not contain any studies with human participants or animals performed by any of the authors.

## Conclusion

The LAMP assay was capable of amplifying a DNA target under isothermal conditions and the amplification products can be quantitatively detected. The assay could be performed in less than 20 minutes without special equipment. In conclusion, this method offered a great advantage for the rapid onsite diagnosis of infection of *Enterocytozoon hepatopenaei* in aquatic products. It can be considered as a powerful technology for the monitoring/tracing of *Enterocytozoon hepatopenaei*, which was rapid, accurate, simple and economic, with good sensitivity and specificity.

## Supplementary information

Supplementary material
