# Supplementary material for: Employing DNA binding dye to improve detection of Enterocytozoon hepatopenaei in real-time LAMP
Source: Sci Rep. 2019 Nov 1;9:15860. doi: 10.1038/s41598-019-52459-0 (PMC6825238; doi:10.1038/s41598-019-52459-0)
Supplement: Supplementary file 1 — Supplementary material [file 41598_2019_52459_MOESM1_ESM.doc]

*Supporting Information*

*For*

Employing DNA binding dye to improve detection of Enterocytozoon hepatopenaei in real-time LAMP

Biao Ma, Huanteng Yu, Jiehong Fang, Chuanxin Sun, Mingzhou Zhang

**Supplementary Figure**


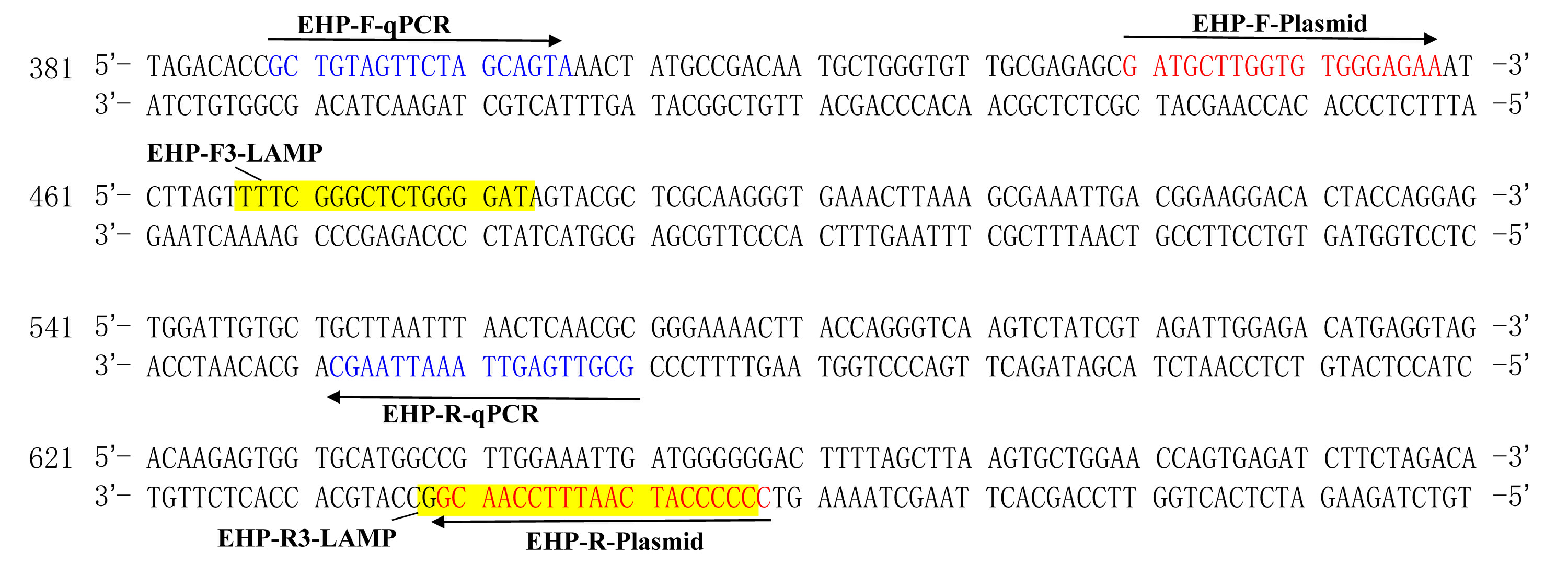


Figure S1: Location of primers for EHP SSU rRNA gene. Partial sequence of SSU rRNA gene and the location of primers, arrows indicate the direction of extension.


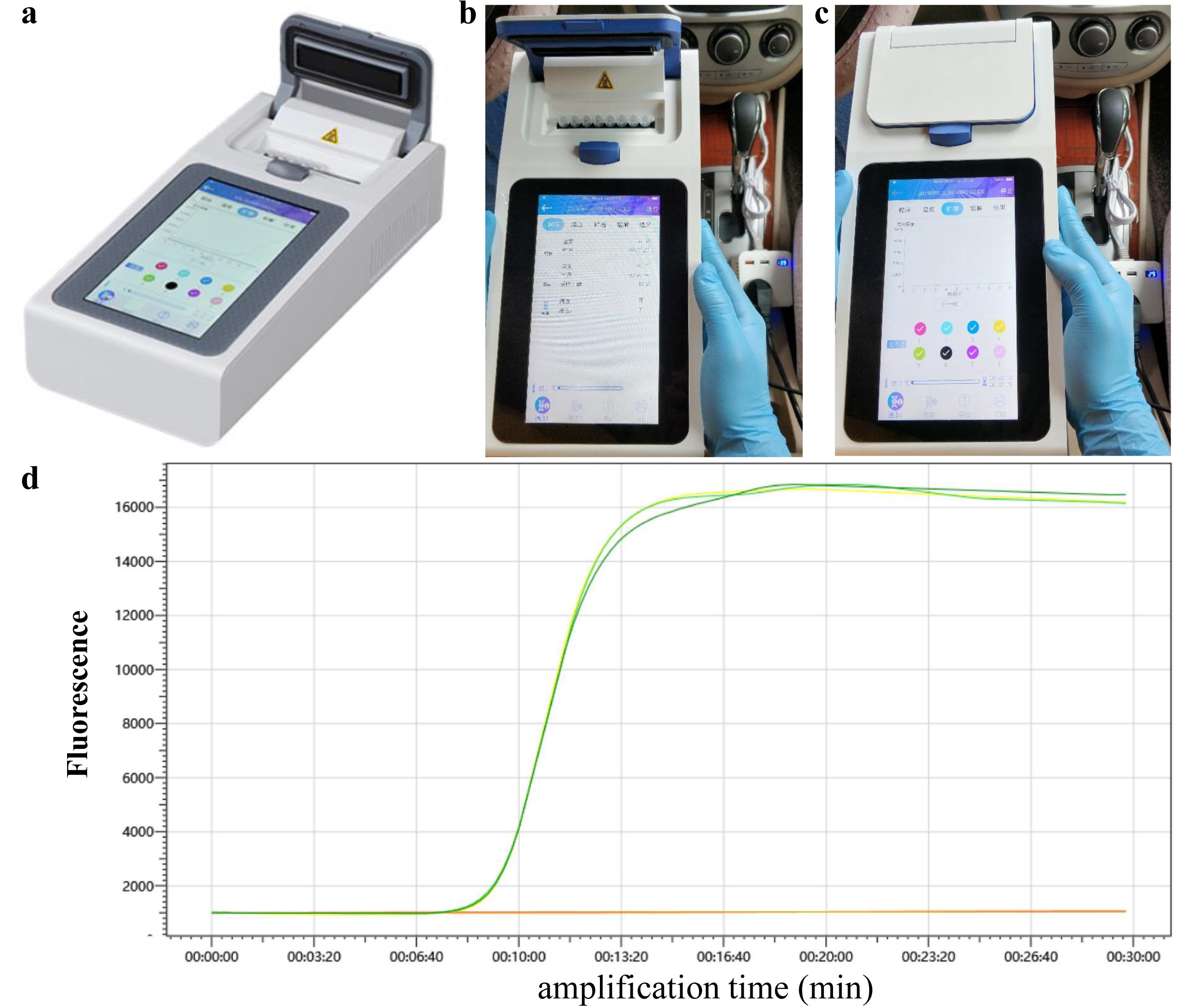


Figure S2: Detection with blind samples by using portable equipment. The quantitative LAMP assay was compatible with deployment to a non-laboratory setting, considering the environment of on-site testing of actual samples. It could be applied on the portable constant temperature fluorescence detecting instrument (Gene-8C, Allsheng Instruments Co. Ltd., Hangzhou, China).

Table S1: The purity of DNA in positive specimens. The data were obtained by the micro-spectrophotometer (Nano100, Allsheng Instruments Co. Ltd., Hangzhou, China).

| Number | concentration (ng/µL) | A260/A280 | A260/A230 | amplification time (min) |
| --- | --- | --- | --- | --- |
| EHP 1-1 | 17.86 | 1.64 | 0.86 | 22.13±0.21 |
| EHP 1-2 | 16.24 | 1.49 | 0.89 | 23.07±0.23 |
| EHP 1-3 | 51.34 | 1.31 | 0.67 | 21.45±0.30 |
| EHP 1-4 | 33.43 | 1.16 | 0.36 | 21.74±0.28 |
| EHP 1-5 | 24.17 | 1.17 | 0.46 | 22.03±0.31 |
| EHP 1-6 | 22.46 | 1.01 | 0.23 | 22.78±0.39 |
| EHP 1-7 | 25.95 | 1.15 | 0.49 | 21.88±0.26 |
| EHP 1-8 | 34.67 | 1.14 | 0.59 | 21.77±0.25 |
| EHP 1-9 | 34.57 | 1.21 | 0.38 | 21.73±0.27 |
| EHP 1-10 | 30.12 | 1.18 | 0.35 | 21.76±0.28 |
| EHP 1-11 | 21.71 | 1.01 | 0.27 | 22.84±0.40 |
| EHP 2-1 | 19.35 | 0.89 | 0.19 | 22.99±0.56 |
| EHP 2-2 | 24.53 | 1.12 | 0.50 | 21.95±0.29 |
| EHP 2-3 | 23.77 | 0.86 | 0.58 | 22.35±0.51 |
| EHP 2-4 | 16.51 | 0.93 | 0.22 | 23.13±0.41 |
| EHP 2-5 | 24.52 | 0.87 | 0.56 | 22.34±0.86 |
| EHP 3-1 | 21.52 | 0.88 | 0.44 | 22.93±0.87 |
| EHP 3-2 | 17.17 | 0.93 | 0.16 | 23.11±0.42 |
| EHP 3-3 | 28.32 | 1.35 | 0.58 | 21.81±0.41 |
| EHP 3-4 | 27.38 | 1.45 | 0.71 | 21.80±0.22 |
| EHP 3-5 | 9.34 | 1.22 | 0.17 | 23.08±0.39 |
| EHP 3-6 | 17.12 | 1.43 | 0.49 | 22.42±0.25 |
| EHP 3-7 | 8.26 | 1.64 | 0.21 | 23.12±0.88 |
| EHP 4-1 | 29.73 | 1.60 | 0.83 | 21.71±0.22 |
| EHP 4-2 | 44.39 | 1.66 | 0.87 | 21.66±0.24 |
| EHP 4-3 | 54.12 | 1.64 | 0.66 | 21.09±0.34 |
| EHP 4-4 | 16.43 | 1.29 | 0.24 | 22.68±0.37 |
| EHP 4-5 | 15.12 | 1.24 | 0.22 | 22.71±0.38 |
| EHP 4-6 | 21.81 | 1.10 | 0.75 | 22.81±0.26 |
| EHP 4-7 | 11.18 | 1.17 | 0.44 | 22.98±0.26 |
| EHP 4-8 | 14.39 | 1.66 | 0.53 | 22.21±0.32 |
| EHP 4-9 | 29.03 | 1.50 | 0.69 | 21.72±0.33 |
| EHP 4-10 | 16.35 | 1.24 | 0.25 | 22.69±0.36 |
| EHP 4-11 | 19.72 | 1.33 | 0.47 | 22.87±0.30 |
